# Supplementary material for: Usp10 Modulates the Hippo Pathway by Deubiquitinating and Stabilizing the Transcriptional Coactivator Yorkie
Source: Int J Mol Sci. 2019 Nov 29;20(23):6013. doi: 10.3390/ijms20236013 (PMC6928647; doi:10.3390/ijms20236013)
Supplement: Supplementary file 1 [file ijms-20-06013-s001.pdf]

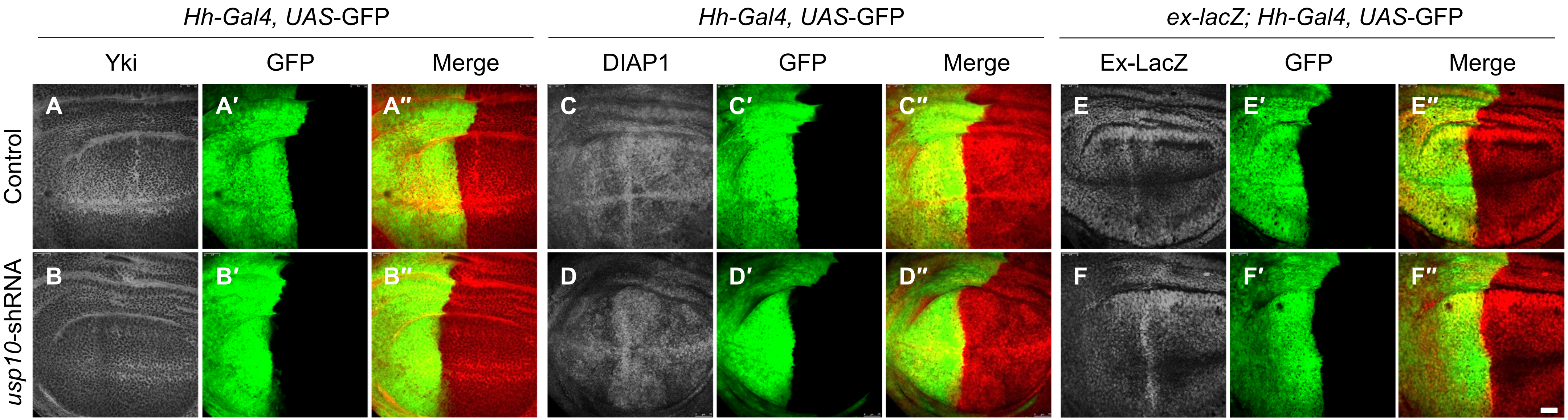

Supplementary Figure 1. *usp10*-shRNA reduces Yki protein level and target genes expression. The following sequences were used to generate *usp10*-shRNA line: Forward primer: 5'-CTAGCAGTGATTTGCAACAATCGTAATAATAGTTATATTCAAGCATATTATTACGATTGTTGCAAATCGCG-3'; Reverse primer: 5'-AATTCGCGATTTGCAACAATCGTAATAATATGCTTGAATATAACTATTATTACGATTGTTGCAAATCACTG-3'.

Shown are wing imaginal discs from third instar larvae with posterior to left and notum up. the scale bars are 50 μm. (A-A″) Representing wing imaginal disc of control (*Hh-Gal4, UAS-GFP*/+) was dissected and immunostained to show the expression patterns of Yki (white in A and red A″) and GFP (green in A′-A″). GFP indicates the expression pattern of Hh-Gal4. (B-B″) Representing wing imaginal disc of *usp10* knockdown (*Hh-Gal4, UAS-GFP*/*UAS-usp10*-shRNA) was dissected and immunostained to show the expression patterns of Yki (white in B and red in B″) and GFP (green in B′-B″). (C-C″ and D-D″) Representing wing imaginal discs of control (*Hh-Gal4, UAS-GFP*/+) (C-C″) and *usp10* knockdown (*Hh-Gal4, UAS-GFP*/*UAS-usp10*-shRNA) (D-D″) were dissected and immunostained to shown the expression patterns of DIAP1 (white in C and D, red in C″ and D″) and GFP (green in C′-C″ and D′-D″). (E-E″ and F-F″) Representing wing imaginal discs of control (*ex-LacZ*/+; *Hh-Gal4, UAS-GFP*/+) (E-E″) and *usp10* knockdown (*ex-LacZ*/+; *Hh-Gal4, UAS-GFP*/*UAS-usp10*-shRNA) (F-F″) were dissected and immunostained to shown the expression patterns of Ex-LacZ (white in E and F, red in E″ and F″) and GFP (green in E′-E″ and F′-F″).

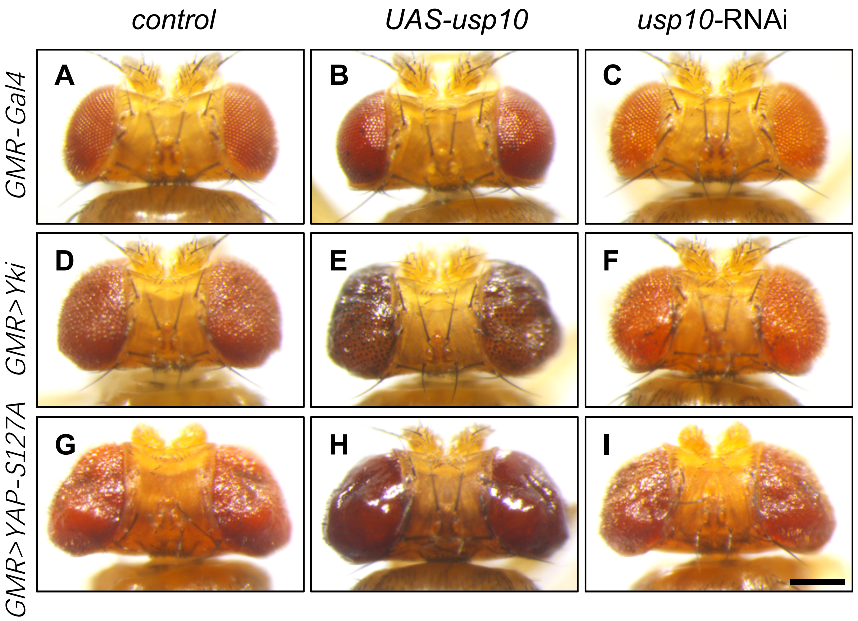

Supplementary Figure 2. Usp10 regulates Yki activity *in vivo.* (A-I) Shown are images for top view of adult male fly eyes, the scale bars are 200 μm. The genotypes are listed*: GMR-Gal4/*+ *(A), GMR-Gal4/*+*, UAS-usp10/*+ (B)*, GMR-Gal4/*+*, UAS-usp10*-RNAi/+ (C), *GMR-Gal4, UAS-Yki/*+ (D), *GMR-Gal4, UAS-Yki/*+*; UAS-usp10/*+ (E), *GMR-Gal4, UAS-Yki/*+*; UAS-usp10*-RNAi/+ (F), *GMR-Gal4, UAS-YAP-S127A/*+ (G), *GMR-Gal4, UAS-YAP-S127A/*+*; UAS-usp10/*+ (H), *GMR-Gal4, UAS-YAP-S127A/*+*; UAS-usp10*-RNAi/+ (I).
